# Oxidative stability and Sensoric acceptability of functional fish meat product supplemented with plant_−_based polyphenolic optimal extracts

**DOI:** 10.1186/s12944-019-0982-y

**Published:** 2019-01-31

**Authors:** Muhammad Ali, Muhammad Imran, Muhammad Nadeem, Muhammad Kamran Khan, Muhammad Sohaib, Hafiz Ansar Rasul Suleria, Reeja Bashir

**Affiliations:** 1 Department of Food Science, Nutrition & Home Economics, Government College University, Faisalabad, Pakistan; 2 Institute of Home and Food Sciences, Faculty of Life Sciences, Government College University, Faisalabad, Pakistan; 3 Department of Dairy Technology, University of Veterinary and Animal Sciences, Lahore, Pakistan; 4 Department of Food Science and Human Nutrition, University of Veterinary and Animal Sciences, Lahore, Pakistan; 5 UQ Diamantina Institute, Faculty of Medicine, Translational Research Institute, The University of Queensland, Brisbane, Australia; 6 Department of Food, Nutrition, Dietetics and Health, Kansas State University, Manhattan, USA; 7 Centre for Chemistry and Biotechnology, School of Life and Environmental Sciences, Deakin University, Melbourne, Vic Australia

**Keywords:** TPC, Ultrasound optimization, Supplementation, Fish meat, Functional foods, Shelf life

## Abstract

**Background:**

Fish meat and its products are usually accepted as good source of biological high value food components and especially for polyunsaturated fatty acids. The quality of fish meat products is considered to be decreased by the lipid peroxidation which leads to reduction in nutritional quality, financial loss and severe health problems. Many tactics are present to reserve their quality and safety. In the present investigation, the extraction and supplementation of optimal total polyphenol extracts (TPC) from vegetable and fruit by_−_products was explored for lipids oxidative stability and sensoric acceptability of functional fish product samples.

**Methods:**

Vegetable and fruit by_−_products (cabbage leaves and banana peels) were collected from local fruits and vegetables processing industries. A 3_−_level five factor Box_−_Behnken design was used to study the effect of extraction/sonication temperature (°C), amplitude level, water/meal ratio, extraction/sonication time (minutes) and pH conditions for maximum yield of TPC from dried vegetable and fruit samples. The TPC samples were analyzed for chemical composition (total polyphenols, cyanogenic contents and tannins). Natural TPC extracts were supplemented at different concentration (0.5, 1 and 1.5%) to fish meat for preparation of different meat ball samples. The fish meat product samples without supplementation of TPC extract were kept as control. The partial/parfrying of the products was carried out to determine the lipid stability (peroxide value and free fatty acids) stored at refrigerator (for 9 days) and at − 18 °C in a freezer for a storage period of 60_−_days. The sensoric analysis (color, flavor and overall acceptability) was performed at different storage intervals for experimental treatments.

**Results:**

The percent values of TPC yield from cabbage leave and banana peel samples ranged from a from minimum value of 9.8 ± 0.12% to a maximum value of 19.8 ± 0.15% for cabbage leaves and minimum value of 15.55 ± 0.13% to a maximum value of 24.4 ± 0.17% for banana peels, respectively. The results revealed that extraction conditions significantly affect the TPC yield from cabbage leaves and banana peels. The cabbage leaves and banana peels contain up to 4.8% total phenolics, cyanogenic compounds (1.44 − 1.47 ± 0.14) and tannins (6.55–7.90 ± 0.22). Peroxide values (meqO_2_ /kg) of meat balls treated with TPC extracts at 4 °C were in the range of 1.31 ± 0.12 to 3.10 ± 0.20 while at − 18 °C ranged was found 1.31 ± 0.12 to 1.55 ± 0.17, respectively. Peroxide values of all the treatments increased at the end of second interval then decreased at the end of last storage interval. Peroxide values of all treatments were higher and significantly different at the beginning and the end of the storage period (*p* <  0.05). In a similar way, free fatty acids and moisture content values trend was recorded for all experimental treatments. Sensory scores of fish product samples for color, flavor and overall acceptability showed a significant difference in sensory scores at refrigeration temperatures where sensory scores of fish product samples decreased significantly (*p* <  0.05) throughout refrigeration storage. Whereas, the sensory scores at the − 18 °C shows the good sensory characteristics, relatively.

**Conclusions:**

Phenolic extracts containing antioxidant status can interact with free lipidperoxy or lipidoxy free radicals (formed in result of lipid oxidation) and hence stopping their further self_−_breakdown. Plant_−_based phenolic extracts can be used to decrease oxidation process and increase the shelf life of fish meat products. Additional studies should be undertaken to determine the maximal shelf life of food products supplemented with different plant_−_based polyphenol extracts and treatment of nutritional disorders through their absorption, metabolism and distribution pattern into biological tissues.

## Background

Meat is an important constituent of a well_−_balanced and healthy diet due to its nutritional status. Some epidemiological data has shown a link between its consumption and increased risk of cardiovascular, metabolic diseases and several forms of cancers [1]. On the other hand, fish meat is considered as abundant source of long chain polyunsaturated fatty acids and valuable nutritional components [2]. However, color and lipid oxidation are main reasons of quality deterioration in the meat products throughout storage which has near about estimate price of the industry loss over $700 million annually. The most instances oxidation is lipid oxidation and it is a free radical chain reaction which can be termed as initiation, propagation, and termination processes [3]. It is a primary mechanism which deteriorates the quality such as development of off_−_flavor, rancidity, degradation in texture, changing of color in meat and its products during storage, which renders them and made meat unfit for human consumption. These reactions take meat towards loss of nutritive value and eventually declining consumer confidence in the product. Intake of such food products which contain lipid constituents which are oxidized can modify proteins, DNA, tumor initiation and membrane structure in biological system [4, 5].

Improved oxidative stability of raw product is well thought_−_out valuable for both the processing industry and the end users. In the vegetable and fruit industry, the procedures of preparation and processing may lead to 1/3rd of the product being rejected. The disposal of by_−_products signifies a rising difficult since the material of plant is typically prone to microbial decay which therefore restrict further exploitation. The residues of fruits and vegetables could be cheap and freely accessible resources of compounds which are bioactive and use in the pharmaceutical and food industries. Diverse antioxidant potency is present in different types of fruit residues and the deviation is very huge [6]. In previous works, the antioxidant strength and amounts of phenolic compounds were noticed to be high in some vegetable and fruit peels which indicates that plant deposits have the ability to be consumed as a source of bioactive compounds e.g. as natural antioxidants [7, 8]. By_−_products of vegetables and fruits are also good supply of micro_−_nutrients which are organic in nature; such as poly_−_phenolics, carotenoids, vitamin C, tocopherols and other bioactive compounds and these compounds can be utilized as food fortification and dietary supplements purposes [9–11].

A sharp responsiveness of customers nowadays is on a relationship between health and diet. The prevention of disease has nurtured growth in the enlargement of ‘functional’ food products. The supplementation of antioxidants in functional diet can protect human body from adverse events and dysfunction of metabolic syndrome because of the beneficial effects of these phytochemicals [12]. Epidemiological work has pointed out that intake of functional foods imparts in health benefits, for example, it reduced the risk of stroke, coronary heart disease as well as certain cancer types [13]. However, available locally and internationally scientific literature does not provide information that consumption of functional meat products supplemented with vegetable and fruit phytochemical extracts are beneficial for health or not. Additionally, research on interactions and stability of phytochemical extracts with meat elements throughout processing process and storage need to be initiated. Therefore, the supplementation study of vegetable and fruit phytochemical extracts in meat products has been targeted in the present investigation which may provide information regarding phytochemicals action to prevent the formation of potential oxidized free fatty acid and peroxide compounds in meat product. In particular, the focus was on the amounts of vegetable and fruit phytochemicals that will not cause any major problems for sensoric acceptability of functional fish meat product.

## Methods

### Extraction of total polyphenols contents (TPC)

The by_−_products (cabbage leaves and banana peels) were produced from fresh vegetable and fruit samples. The peel samples were thoroughly washed under tap water to remove dirt, dust, microflora and pesticide residue on the surface. After that, the peel samples were further cut into small pieces using a stainless steel knife and oven dried at 50 °C for 48 h in hot air oven until moisture content fell below 10%. Dried peels were ground to fine powder through the sample mill with sieve size 0.5 mm. The peel powder was weight (100 ± 0.1 g) using the electronic weighing balance (Model Kern 440_−_ 35 N) for each treatment. A 3_−_level five factor Box_−_Behnken design was used to study the effect of extraction/sonication temperature (°C), amplitude level, water/meal ratio, extraction/sonication time (minutes) and pH conditions for maximum yield of total polyphenols from dried vegetable and fruit samples.

The percent yield of peel polyphenol extract was assessed by dividing the weight of the extract with the sample weight and multiplying by 100. The coded design of real experiments is given in Table 1. The pH of the solution was monitored continuously and was adjusted by 0.2 mol/L NaOH and HCl, respectively while the temperature of the aqueous system was controlled within ±1.5 °C. The extracted TPC solutions were separated and then dried in a hot air oven at 50 °C. Yield of TPC was calculated as percentage of dried polyphenol extract.

**Table 1:** Coded and actual levels of independent variables for optimization of total polyphenol extraction yield as determined by Box_−_Behnken design

Independent Variables	Coded Levels		
	–1	0	+ 1
Extraction Temperature (°C)	40	50	60
Amplitude Level (%)	30	60	90
Water/meal ratio	20	30	40
Extraction Time (Minutes)	20	40	60
Extraction pH	4	6	8

The following empirical “black box” modeling presents the relationships among process and response variables

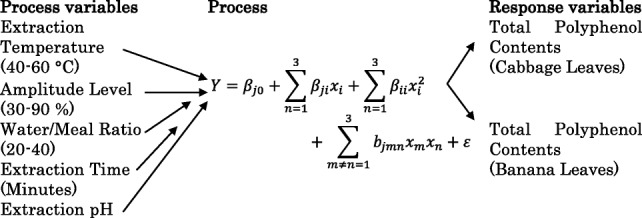



The expression inside the “black box” represents the total polyphenol contents in cabbage and banana leaves when the value of *j* is changed from 1 to 5. The β*jo*; β*ji*; β*jii*; and β*jmn* represent the constant and coefficients of linear, quadratic and interactive effects, respectively. The *x*_*i*_; ${x}_i^2$ and *x*_*m*_*x*_*n*_ represent the linear, quadratic and interactive effects of the independent variables, respectively, and ε is the random error primarily to account for the inability to determine the true model.

### Analysis of TPC extracts

The phytochemical extracts were analyzed for chemical composition. Total polyphenols were measured by using Folin_−_Ciocalteu method following the protocol of Singleton et al. [14]. The cyanogenic contents in extract samples were estimated by alkaline titration according to the method outlined in AOAC [15] Method No. 26.115. For tannins determination, at 700 nm through spectrophotometer, the concentrations of each sample were used [16].

### Functional meat product development and analysis

Fresh fish rohu (*Labeo rohita*) was obtained from fish supermarket, Punjab, Pakistan. Natural vegetable and fruit phytochemical extracts were supplemented at different concentration (0.5, 1 and 1.5%) to fish meat for preparation of different meat balls. The fish meat product samples without supplementation of TPC extract were kept as control. The partial/parfrying of the products was carried out to determine the lipid stability. Fried meat product samples were vacuum sealed in plastic bags and then were stored at refrigerator (for 9 days) and at − 18 °C in a freezer for a storage period of 60_−_days. The moisture content and also the oxidative stability of oils extracted from fish meat products was assessed by measuring peroxide value (AOCS Method No. Cd 8–53) and free fatty acid value (AOCS Method No. Ca 5a–40), respectively [17].

### Sensoric evaluation

Experienced and untrained assessors carried out the sensory analysis of meat product samples according to the instructions given by Meilgaard et al. [18]. Each judge gave the written informed consent after explanation of risks and benefits of participation prior to the study. The panelists were provided informative instructions and brief definitions of attributes such as color, flavor and overall acceptability. Samples were presented to participants in sensory booths under white lighting. The order of presentation was balanced to avoid carry_−_over effects. Each panelist received the samples assigned with random three–digit code numbers. Each panelist was asked to list their preference on a 9–cm comparison line (1 = dislike extremely to 9 = like extremely). The sensoric analysis was performed at different storage intervals for experimental treatments.

### Statistical analysis

The behavior of the Box_−_Behnken Model was explained by the following the quadratic equation. The data of TPC yield obtained for each treatment was subjected to statistical analysis to determine the level of significance by using the software package MATLAB according to the method described by Montgomery [19]. The average of the three runs was reported as the measured value with standard deviation. The sample analysis for storage stability and consumer acceptability was carried out in triplicate and the significant differences were calculated among means at a probability level of 5%.

## Results and discussion

### Optimal extraction of TPC

A 3_−_level five factors Box_−_Behnken design was used to study the effect of extraction temperature (°C), amplitude power level, water/meal ratio, extraction time (minutes) and extraction pH conditions for maximum yield of total polyphenols from cabbage leaves and banana peels. The conditions of response surface experiment were determined by setting the extraction temperature (°C), amplitude level (%), water/meal ratio, extraction time (minutes) and pH conditions on standardizing levels from − 1 to + 1 for each single factor. The total number of experimental runs for each cabbage leaves and banana peels was 46 as determined by the Box_−_Behnken design. The experimental response regarding TPC yield in cabbage leave and banana peel samples as a result of different extraction treatments have been depicted in Table 2. The percent values of TPC yield from cabbage leave and banana peel samples ranged from a from minimum value of 9.8 ± 0.12% to a maximum value of 19.8 ± 0.15% for cabbage leaves and minimum value of 15.55 ± 0.13% to a maximum value of 24.4 ± 0.17% for banana peels, respectively. The results revealed that extraction conditions significantly affect the TPC yield from cabbage leaves and banana peels. There is very limited published data that provides an information or support to the TPC yield from cabbage leaves and banana peels. The extraction run point 25 (extraction temperature, 60 °C; amplitude/sonication level, 90; extraction pH, 6; water/meal ratio, 30 and extraction time, 40 min) showed maximum TPC yield for cabbage leaves (19.8 ± 0.15%) and banana peel (24.4 ± 0.17%), respectively and the lowest TPC yield 15.55 ± 0.13% and 9.8 ± 0.12% was for extraction run 4 (extraction temperature, 50 °C; extraction pH, 4; water/meal ratio, 30; amplitude level 30 and extraction time, 40 min) of banana peels and extraction run 5 (extraction temperature, 40 °C; extraction pH, 6; water/meal ratio, 30; amplitude level 30 and extraction time, 40 min) of cabbage leaves, respectively.

**Table 2:** Percent values of total polyphenol extract yield as determined by the Box_−_Behnken design

Extraction run	Independent Variables					Total Polyphenols Extract Yield (%)	
	Extraction Temperature (°C)	Amplitude Level (%)	Water/meal ratio	Extraction Time (Minutes)	Extraction pH	Cabbage Leaves (R_1_)	Banana Peels (R_2_)
1	50 (0)	60 (0)	40 (+ 1)	40 (0)	8(+ 1)	18.55	23.05
2	60 (+ 1)	60 (0)	30 (0)	40 (0)	8 (+ 1)	19.82	24.34
3	50 (0)	60 (0)	20 (−1)	40 (0)	4(− 1)	12.95	17.45
4	50 (0)	30 (− 1)	30 (0)	40 (0)	4 (−1)	11.05	15.55
5	40 (−1)	30 (− 1)	30 (0)	40 (− 1)	6 (0)	9.80	14.31
6	50 (0)	30 (−1)	30 (0)	60 (+ 1)	6 (0)	13.62	18.15
7	50 (0)	60 (0)	30 (0)	20 (−1)	4 (−1)	12.85	17.35
8	50 (0)	30 (−1)	20 (− 1)	40 (0)	6 (0)	11.70	16.22
9	40 (−1)	60 (0)	30 (0)	40 (0)	8 (+ 1)	14.82	19.34
10(C_1_)	50 (0)	60 (0)	30 (0)	40 (0)	6 (0)	14.85	19.33
11	40 (−1)	60 (0)	30 (0)	60 (+ 1)	6 (0)	13.60	18.11
12	50 (0)	60 (0)	40 (+ 1)	60 (+ 1)	6 (0)	17.34	21.80
13	60 (+ 1)	30 (−1)	30 (0)	40 (0)	6 (0)	14.85	19.34
14	60 (+ 1)	60 (0)	40 (+ 1)	40 (0)	6 (0)	18.50	23.02
15	50 (0)	30 (−1)	30 (0)	40 (0)	8 (+ 1)	14.84	19.36
16	60 (+ 1)	60 (0)	30 (0)	20 (−1)	6 (0)	16.60	21.12
17	50 (0)	60 (0)	40 (+ 1)	20 (−1)	6 (0)	15.30	19.80
18	50 (0)	90 (+ 1)	30 (0)	60 (+ 1)	6 (0)	18.62	23.13
19	50 (0)	60 (0)	30 (0)	60 (+ 1)	4 (−1)	14.85	19.35
20	50 (0)	60 (0)	20 (−1)	40 (0)	8 (+ 1)	16.70	21.20
21	40 (−1)	60 (0)	40 (+ 1)	40 (0)	6 (0)	13.51	18.04
22(C_2_)	50 (0)	60 (0)	30 (0)	40 (0)	6 (0)	14.80	19.37
23	50 (0)	30 (−1)	30 (0)	20 (−1)	6 (0)	11.60	16.10
24	50 (0)	60 (0)	20 (−1)	20 (− 1)	6 (0)	13.50	18.02
25	60 (+ 1)	90 (+ 1)	30 (0)	40 (0)	6 (0)	19.82	24.34
26	50 (0)	60 (0)	30 (0)	60 (+ 1)	8 (−1)	18.60	23.12
27	50 (0)	30 (−1)	40 (+ 1)	40 (0)	6 (0)	13.50	18.04
28	50 (0)	90 (+ 1)	30 (0)	20 (−1)	6 (0)	16.65	21.16
29	50 (0)	90 (+ 1)	30 (0)	40 (0)	6 (0)	14.83	19.34
30	50 (0)	60 (0)	20 (−1)	40 (0)	6 (0)	16.70	21.22
31	50 (0)	90 (+ 1)	20 (−1)	60 (+ 1)	6 (0)	15.50	20.04
32(C_3_)	50 (0)	60 (0)	30 (0)	40 (0)	6 (0)	14.81	19.31
33	40 (−1)	60 (0)	20 (− 1)	40 (0)	6 (0)	11.72	16.22
34	60 (+ 1)	60 (0)	30 (0)	60 (+ 1)	6 (0)	18.60	23.10
35	60 (+ 1)	60 (0)	30 (0)	40 (0)	4 (−1)	16.05	20.55
36	40 (−1)	90 (+ 1)	30 (0)	40 (0)	6 (0)	14.80	19.30
37	40 (−1)	60 (0)	30 (0)	20 (−1)	6 (0)	12.04	17.05
38(C_4_)	50 (0)	60 (0)	30 (0)	40 (0)	6 (0)	14.82	19.34
39	50 (0)	90 (+ 1)	30 (0)	40 (0)	8 (+ 1)	19.85	24.36
40	40 (−1)	60 (0)	30 (0)	40 (0)	4 (−1)	11.05	15.55
41	50 (0)	60 (0)	40 (+ 1)	40 (0)	4 (−1)	14.75	19.25
42(C_5_)	50 (0)	60 (0)	30 (0)	40 (0)	6 (0)	14.83	19.39
43	60 (+ 1)	60 (0)	20 (−1)	40 (0)	6 (0)	16.72	21.20
44	50 (0)	90 (+ 1)	40 (+ 1)	40 (0)	6 (0)	10.50	23.04
45	50 (0)	60 (0)	30 (0)	20 (−1)	8 (+ 1)	16.60	21.10
46	50 (0)	90 (+ 1)	30 (0)	40 (0)	4 (−1)	16.05	20.55

^C1,C2,C3,C4,C5^represent extraction process at center points

R_1_ and R_2_ represent response factors

The TPC yield obtained from cabbage leaves (15.55 ± 0.13%) and banana peels (24.4 ± 0.17%) was similar to findings of earlier reported studies in literature. Although extraction yields obtained were similar or higher to those described in the literature and former methods of extraction were more aggressive than the proposed in the present work. The results indicated that TPC yield was significantly affected by the model effects (Table 3) of cabbage leaves and banana peels, respectively. The linear and quadratic effects model effects were observed more significant as compared to interaction (*p* ≤ 0.01). The order of model effects observed during TPC yield from dried cabbage leaves and banana peels was linear>quadratic>interaction. The results further substantiated that the linear coefficients (extraction temperature, extraction pH, amplitude level and extraction time) (*p* ≤ 0.01) and quadratic term coefficient (extraction pH × extraction pH) (water/meal ratio × water/meal ratio) (*p* ≤ 0.01) were most significant.

**Table 3:** ANOVAs of the predicted second_−_order polynomial model for total polyphenol extract yield of cabbage leaves and banana peels

Source of Variation		df	Cabbage Leaves		Banana Peels	
			F value	*p* value	F value	*p* value
Linear	Intercept	20	4482.57***	< 0.0001	442.02***	<0.0001
	A:Extraction Temperature	1	30,628.13***	< 0.0001	33,065.09***	<0.0001
	B:Amplitude Level	1	31,250.00***	< 0.0001	3095.98***	<0.0001
	C:Water/meal ratio	1	4050.00***	< 0.0001	401.24***	<0.0001
	D:Extraction Time	1	4753.13***	< 0.0001	401.24***	<0.0001
	E:Extraction pH	1	17,578.13***	< 0.0001	1741.49***	<0.0001
Interaction	AB	1	0.000	1.0000	0.000	1.0000
	AC	1	0.000	1.0000	0.000	1.0000
	AD	1	12.50	0.0016	0.31	1.5829
	AE	1	0.000	1.0000	0.000	1.0000
	BC	1	0.000	1.0000	0.000	1.0000
	BD	1	0.000	1.0000	0.000	1.0000
	BE	1	0.000	1.0000	0.000	1.0000
	CD	1	0.000	1.0000	0.000	1.0000
	CE	1	0.000	1.0000	0.000	1.0000
	DE	1	0.000	1.0000	0.000	1.0000
Quadratic	A^2^	1	1.70	0.2036	2.70	< 0.1127
	B^2^	1	0.19	0.6672	0.30	< 0.5886
	C^2^	1	232.01	< 0.0001	19.21	< 0.0002
	D^2^	1	288.07	< 0.0001	43.23	< 0.0001
	E^2^	1	1037.12	< 0.0001	94.59	< 0.0001
Residual		25	–	–	–	–
Lack of Fit		20	–	–	–	–
Pure Error		5	–	–	–	–
Cor. Total		45	–	–	–	–

***Significant at 0.05 level

^NS^ Non_−_Significant

**Fig. 1:**
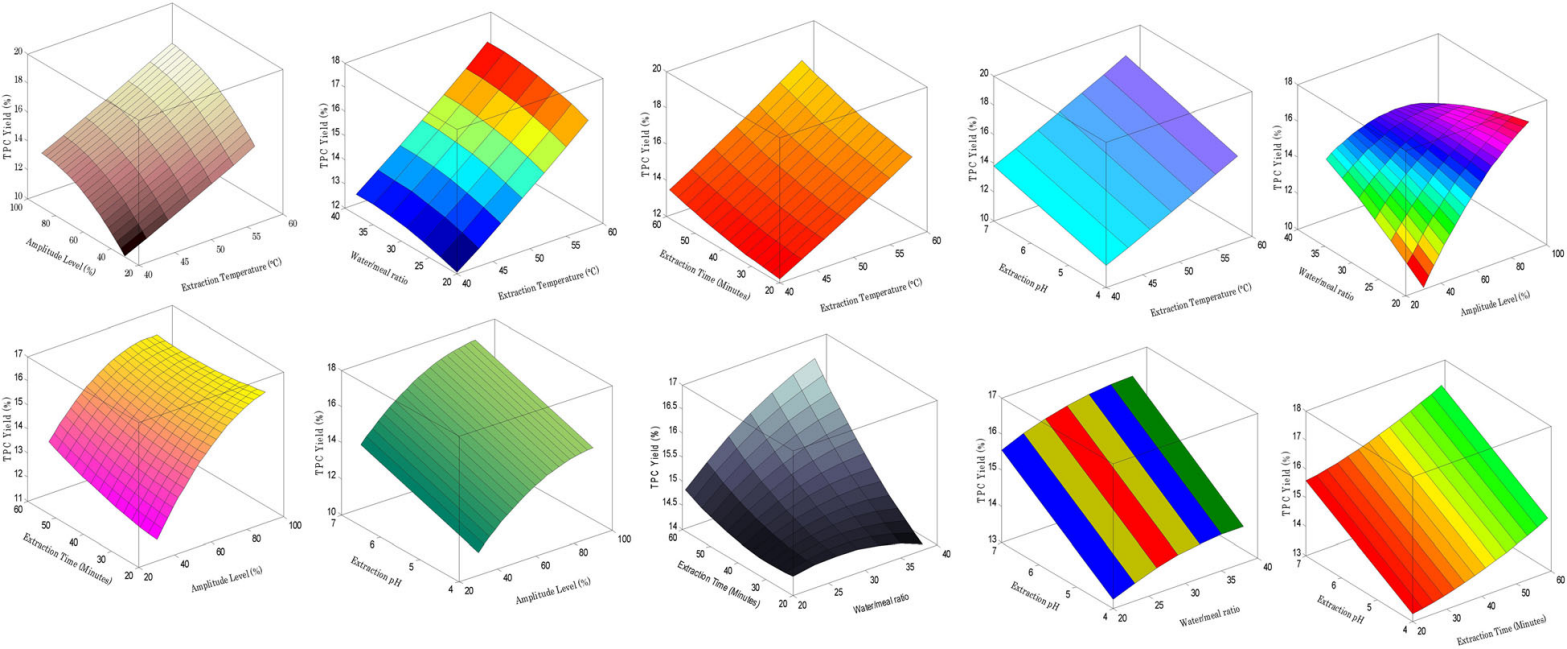
Response surfaces for the mutual interaction effect of ultrasound_−_assisted extraction conditions on the yield of total polyphenol extracts in cabbage leaves

**Fig. 2:**
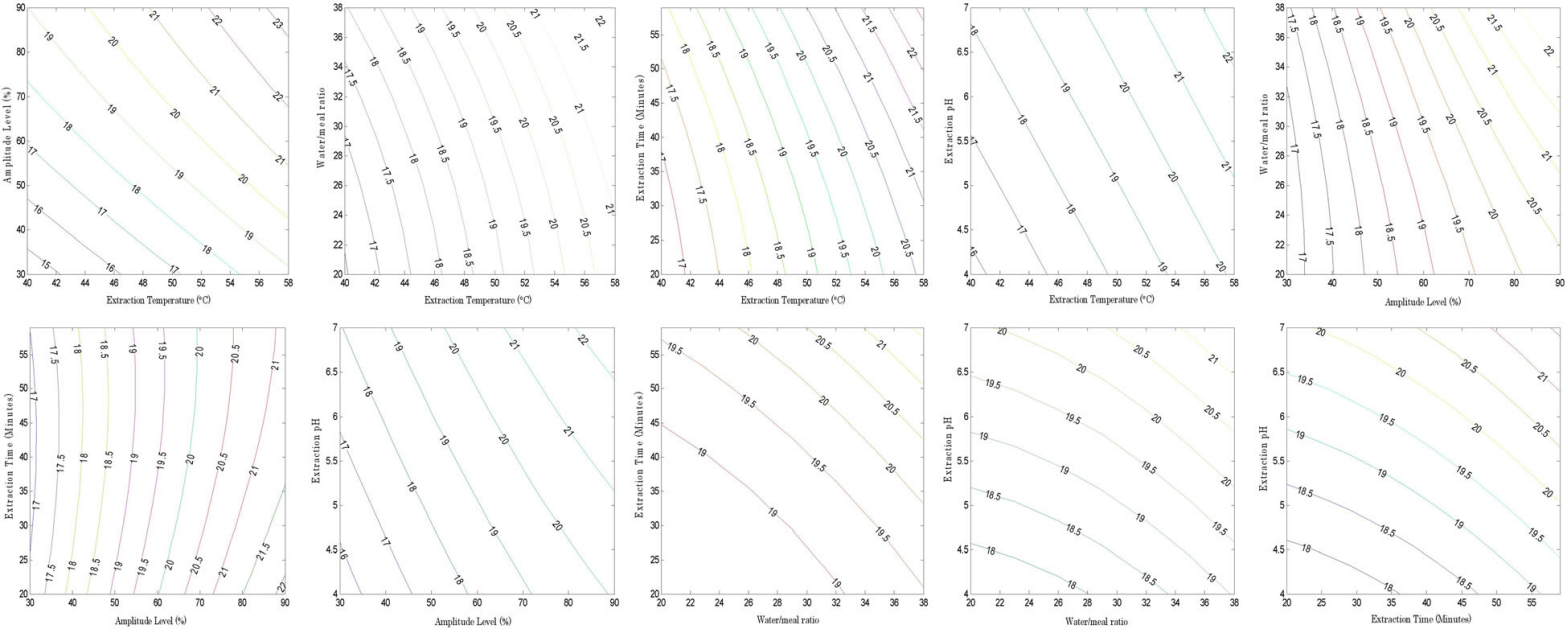
Contour plots for the mutual interaction effect of extraction conditions on the yield of total polyphenol extracts in banana peels

The ultimate objective of employing response surface methodology in this study was to find out the significant effects of parameters viz., extraction temperature, amplitude level, water/meal ratio, pH and extraction/sonication time to find out the optimum conditions for TPC yields. The conditions and mutual interaction terms which determine the rate of maximum TPC yields are difficult to optimize and cannot be manipulated directly from the response model. The validated optimum extraction conditions for the maximum TPC yields were obtained by varying two independent parameters and fixing the other three variables at the coded zero level (Fig. 1 and Fig. 2). The results showed the temperature was major effective factor. It has also been cited that increase in TPC yield is due to the strong effect of extraction time–temperature on the mass transfer rate of the water_−_soluble polyphenols in the cell wall. However, when extraction temperature and time were kept constant, increase in water:meal ratio was the reason for an exponential increase in the yield. This is due to the availability of more liquid which increases the driving force of polyphenols out of the meal. The researchers showed that the use of the higher temperature is unpractical, because it effects on decreasing resultant ingredient yield of the extract. It was also unpractical to use the meal to water ratio more than optimum, because under these conditions the yield of solids increases not much, but dilution of the system occurs, that reduces its functional properties during further application in food products [20].

### Chemical characterization of TPC extracts

The cabbage leaves and banana peels contained up to 4.8% total phenolics which were very close to the findings of Wadhwa and Bakshi [21]. The cyanogenic compounds (1.44–1.47 ± 0.14) and tannins (6.55–7.90 ± 0.22) mostly present in the by_−_product extracts were found responsible for the astringent taste [22].

### Peroxide values of functional fish meat product

Table 4 presents the mean values of peroxides in different TPC supplemented fish meat product samples stored at temperatures 4 °C and − 18 °C, respectively. Peroxide values (meqO_2_ /kg) of meat balls treated with TPC extracts at 4 °C were in the range of 1.31 ± 0.12 to 3.10 ± 0.20 while at − 18 °C ranged was found 1.31 ± 0.12 to 1.55 ± 0.17, respectively. Peroxide values (PV) of all the treatments increased at the end of second interval then decreased at the end of last storage interval. Peroxide values of all treatments were higher and significantly different at the beginning and the end of the storage period (*p* <  0.05). The decrease of the PV at the end of the storage may occur owing to decomposition of hydro peroxides into secondary oxidation products [23]. A similar trend was also observed by the Yerlikaya et al. [24] during the refrigerated studies of fish patties. The values of peroxide value, free fatty acid, thiobarbutric acid and total volatile base nitrogen in fish burgers at the end of storage increased significantly determined as 4.98 (±0.22) meqO_2_/kg of fat, 0.94 (±0.01) % of oleic acid, 0.58 (± 0.02) mg MA/kg of sample and 4.78 (±0.02) mg/100 g of sample, respectively [23]. Several lipid oxidation indices were assessed to follow up the development of oxidation in frozen state. Peroxide value and thiobarbituric acid reactive substances showed primary and secondary oxidation, respectively [25]. When the peroxide value exceeded 10 meq oxygen/ kg fat of meat, the meat is then considered unfit for human consumption or refused [26].

**Table 4:** Peroxide value (meqO_2_ /kg) of meat balls treated with total polyphenol extracts

Treatment	Cabbage Leaves Extracts				Banana Peel Extracts				Cabbage Leaves Extracts				Banana Peel Extracts			
	4 °C				4 °C				−18 °C				− 18 °C			
	0 day	3 days	6 days	9 days	0 day	3 days	6 days	9 days	0 day	15 days	30 days	60 days	0 day	15 days	30 days	60 days
Control	1.35 ± 0.14^g^	2.02 ± 0.18^c^	3.10 ± 0.20^a^	2.07 ± 0.19^b^	1.34 ± 0.14^g^	2.10 ± 0.18^c^	2.90 ± 0.20^a^	2.60 ± 0.19^b^	1.35 ± 0.14^g^	1.44 ± 0.16^e^	1.55 ± 0.17^d^	1.39 ± 0.15^f^	1.34 ± 0.14^g^	1.43 ± 0.16^e^	1.54 ± 0.17^d^	1.38 ± 0.15^f^
0.5%	1.34 ± 0.15^f^	1.85 ± 0.20^a^	1.62 ± 0.19^b^	1.50 ± 0.18^c^	1.33 ± 0.15^f^	1.84 ± 0.20^a^	1.61 ± 0.19^b^	1.49 ± 0.18^c^	1.34 ± 0.15^f^	1.38 ± 0.16^e^	1.41 ± 0.17^d^	1.41 ± 0.17^d^	1.33 ± 0.15^f^	1.37 ± 0.16^e^	1.40 ± 0.17^d^	1.40 ± 0.17^d^
1%	1.33 ± 0.12^e^	1.37 ± 0.14^c^	1.57 ± 0.15^a^	1.48 ± 0.14^b^	1.32 ± 0.17^d^	1.36 ± 0.18^c^	1.56 ± 0.20^a^	1.47 ± 0.19^b^	1.33 ± 0.12^e^	1.35 ± 0.13^d^	1.37 ± 0.14^c^	1.36 ± 0.14^c^	1.32 ± 0.17^d^	1.34 ± 0.18^c^	1.36 ± 0.18^c^	1.35 ± 0.18^c^
1.5%	1.32 ± 0.12^d^	1.35 ± 0.13^c^	1.43 ± 0.15^a^	1.39 ± 0.14^b^	1.31 ± 0.12^d^	1.34 ± 0.13^c^	1.42 ± 0.15^a^	1.38 ± 0.14^b^	1.32 ± 0.12^d^	1.33 ± 0.12^d^	1.35 ± 0.13^c^	1.35 ± 0.13^c^	1.31 ± 0.12^d^	1.32 ± 0.12^d^	1.34 ± 0.13^c^	1.34 ± 0.13^c^

^a-g^Means with different superscripts within an extract category differ significantly (*p* ≤ 0.05)

Decrease of PV could be attributed to different rates of lipid oxidation [27]. Lipids in cooked meat could be more easily oxidized than those in raw meat [28]. The chemical spoilage associated with fish during storage is mainly due to fish lipid degradation (auto_−_oxidation). In general, fish have high degree of unsaturated lipids than other food commodities. Fish lipids are subjected to two main changes, lipolysis and auto_−_oxidation. The main reactants in these processes involves atmospheric oxygen and fish unsaturated lipids, leading to the formation of hydroperoxides, associated with tasteless, flavor and accompanied by brown yellow discoloration of the fish tissue. Upon further degradation of hydroperoxides is the formation of strong rancid flavors e.g. aldehydes and ketones, usually associated with spoilt fatty fish species [26]. The increase of lipid content than the lipid oxidations resulting from action of lipolytic enzymes (lipases and phospholipases) that fish phospholipids undergo degradation to produce hydroperoxides, aldehydes and ketones which are responsible for the development of oxidative rancidity [26]. Oxidation of highly unsaturated lipids is other factor which highly related to the production of off_−_flavors and odors and also as influencing, protein denaturation and texture changes [29]. The most common cause of oil deterioration is rancidity and the most common cause of rancidity in oils and fats is oxidation. Oxidation of the oil, in oily fish, gives rise to rancid odors and flavors; these can limit the storage life of such species more quickly than the protein changes that govern the extractable protein value. An important stage in the oxidation is the reaction of oxygen with the unsaturated fatty acid molecules to form hydroperoxides and the amount of these can be used as a measure of the extent of oxidation in the early stages. The peroxide test is a measure of the formation of hydroperoxides. An increase in the PV is most useful as an index of the earlier stages of oxidation which on oxidation proceeds and peroxides decrease at final stages and the PV can start to fall [24]. Throughout processing and storage of meat, there are three mechanisms for spoilage of meat and its products as A) autolytic enzymatic spoilage, (B) microbial spoilage and (C) lipid oxidation [30]. A number of hypotheses are associated with the disease caused by the consumption of such rancid meat and a meat product includes cardiovascular diseases, cancers of several types and diabetes [31]. Prolong refrigeration of meat and its product can lead to poor quality due to lipid peroxidation. Antioxidants are being used to decrease oxidation process and increase shelf life [32]. Researchers have shown that lipid oxidation can be delayed or inhibit by synthetic or natural food additives e.g. anti_−_oxidants [33]. Phenolic antioxidants interact with free lipidperoxy or lipidoxy free radicals (formed in result of lipid oxidation), hence stopping their further self_−_breakdown. Lipid auto_−_oxidation inhibition is essential not only in foodstuffs during their heating or storage, but also to reduce lipids oxidation after ingestion and absorption via the intestinal wall [34].

### Free fatty acids of functional fish meat product

Table 5 presents the mean values of free fatty acids (FFA) in different TPC supplemented fish product samples stored at temperatures 4 °C and − 18 °C. Free fatty acids of fish products at different storage days was found in parallel to the studies of Vanitha et al. [23]. A similar trend was also observed by the Yerlikaya et al. [24] during the refrigerated studies of fish patties. Genuine FFA contents of lipids from product samples increased in a similar fashion during storage at − 18 °C, while concomitantly their *P* levels decreased [35]. When fish_−_based products are frozen and cold stored, unfavorable changes take place in the texture and appearance. Simultaneously, free fatty acids are formed from the lipids. These changes have been attributed to enzymic reactions which take place at a rate governed by the temperature of frozen storage. The FFA generated during storage originated from the phospholipids, the neutral lipids or both. This is in contrast to the mince lipids where the contributions from both phospholipids and neutral lipids to FFA formation could be calculated [35]. Increase in FFA results from the enzymatic hydrolysis of esterified lipids [36]. Free fatty acids content has been used to establish the grade of deterioration. Lipid (glycerol_−_fatty acids esters) present in the fish muscle undergoes hydrolysis, resulting in the release of fatty acids. Due to lipid hydrolysis, FFA accumulates in the tissue during frozen storage, especially at high temperatures around − 10 °C. Slow freezing rates or fluctuating storage temperatures may result in the lysis of lysosomes and thereby increased activity of some endogenous lipases resulting in increased rates of FFA accumulation [26].

**Table 5:** Free fatty acids (% of oleic acid) of meat balls treated with total polyphenol extracts

Treatment	Cabbage Leaves Extracts				Banana Peel Extracts				Cabbage Leaves Extracts				Banana Peel Extracts			
	4 °C				4 °C				−18 °C				−18 °C			
	0 day	3 days	6 days	9 days	0 day	3 days	6 days	9 days	0 day	15 days	30 days	60 days	0 day	15 days	30 days	60 days
Control	0.21 ± 0.15^f^	0.31 ± 0.17^d^	0.54 ± 0.19^b^	0.63 ± 0.20^a^	0.20 ± 0.15^f^	0.30 ± 0.17^d^	0.53 ± 0.19^b^	0.62 ± 0.20^a^	0.21 ± 0.15^f^	0.27 ± 0.16^e^	0.33 ± 0.17^d^	0.35 ± 0.18^c^	0.20 ± 0.15^f^	0.26 ± 0.16^e^	0.32 ± 0.17^d^	0.34 ± 0.18^c^
0.5%	0.20 ± 0.15^f^	0.30 ± 0.17^d^	0.50 ± 0.19^b^	0.57 ± 0.20^a^	0.19 ± 0.15^f^	0.29 ± 0.17^d^	0.49 ± 0.19^b^	0.56 ± 0.20^a^	0.20 ± 0.15^f^	0.26 ± 0.16^e^	0.28 ± 0.17^d^	0.31 ± 0.18^c^	0.19 ± 0.15^f^	0.25 ± 0.16^e^	0.27 ± 0.17^d^	0.30 ± 0.18^c^
1%	0.22 ± 0.15^f^	0.29 ± 0.17^d^	0.47 ± 0.19^b^	0.51 ± 0.20^a^	0.21 ± 0.15^f^	0.28 ± 0.17^d^	0.46 ± 0.19^b^	0.50 ± 0.20^a^	0.20 ± 0.15^f^	0.25 ± 0.16^e^	0.27 ± 0.17^d^	0.29 ± 0.18^c^	0.19 ± 0.15^f^	0.24 ± 0.16^e^	0.26 ± 0.17^d^	0.28 ± 0.18^c^
1.5%	0.19 ± 0.15^f^	0.27 ± 0.17^d^	0.41 ± 0.19^b^	0.49 ± 0.20^a^	0.18 ± 0.15^f^	0.26 ± 0.17^d^	0.40 ± 0.19^b^	0.48 ± 0.20^a^	0.19 ± 0.15^f^	0.24 ± 0.16^e^	0.26 ± 0.17^d^	0.28 ± 0.18^c^	0.18 ± 0.15^f^	0.23 ± 0.16^e^	0.25 ± 0.17^d^	0.27 ± 0.18^c^

^a-f^Means with different superscripts within an extract category differ significantly (*p* ≤ 0.05)

### Moisture content of functional fish meat product

Table 6 presents the mean values of moisture in different TPC supplemented fish products stored at temperatures 4 °C and − 18 °C. Moisture content of fish products at refrigerated temperature was found in parallel to the studies of Vanitha et al. [23]. While the moisture content of fish products at deep storage was found in parallel to the studies of Mahmoudzadeh et al. [37]. Moisture content increased in all treatments at the end of research trial [37] which is in close to the present findings.

**Table 6:** Moisture content (%) of meat balls treated with total polyphenol extracts

Treatment	Cabbage Leaves Extracts				Banana Peel Extracts				Cabbage Leaves Extracts				Banana Peel Extracts			
	4 °C				4 °C				−18 °C				−18 °C			
	0 day	3 days	6 days	9 days	0 day	3 days	6 days	9 days	0 day	15 days	30 days	60 days	0 day	15 days	30 days	60 days
Control	66.54 ± 0.19^b^	65.59 ± 0.18^c^	65.16 ± 0.17^d^	64.23 ± 0.16^e^	66.53 ± 0.19^b^	65.58 ± 0.18^c^	65.15 ± 0.17^d^	64.22 ± 0.16^e^	66.54 ± 0.19^b^	66.53 ± 0.19^b^	66.66 ± 0.20^a^	66.67 ± 0.20^a^	66.53 ± 0.19^b^	66.52 ± 0.19^b^	66.65 ± 0.20^a^	66.66 ± 0.20^a^
0.5%	66.17 ± 0.19^b^	65.21 ± 0.18^c^	65.09 ± 0.17^d^	64.15 ± 0.16^e^	66.16 ± 0.19^b^	65.20 ± 0.18^c^	65.08 ± 0.17^d^	64.14 ± 0.16^e^	66.17 ± 0.19^b^	66.16 ± 0.19^b^	66.27 ± 0.20^a^	66.28 ± 0.20^a^	66.16 ± 0.19^b^	66.15 ± 0.19^b^	66.26 ± 0.20^a^	66.27 ± 0.20^a^
1%	66.10 ± 0.19^b^	65.14 ± 0.18^c^	65.07 ± 0.17^d^	64.10 ± 0.16^e^	66.09 ± 0.19^b^	65.13 ± 0.18^c^	65.06 ± 0.17^d^	64.09 ± 0.16^e^	66.10 ± 0.19^b^	66.09 ± 0.19^b^	66.19 ± 0.20^a^	66.20 ± 0.20^a^	66.09 ± 0.19^b^	66.08 ± 0.19^b^	66.18 ± 0.20^a^	66.19 ± 0.20^a^
1.5%	66.05 ± 0.19^b^	65.09 ± 0.18^c^	65.01 ± 0.17^d^	64.05 ± 0.16^e^	66.04 ± 0.19^b^	65.08 ± 0.18^c^	65.00 ± 0.17^d^	64.04 ± 0.16^e^	66.05 ± 0.19^b^	66.04 ± 0.19^b^	66.14 ± 0.20^a^	66.15 ± 0.20^a^	66.04 ± 0.19^b^	66.03 ± 0.19^b^	66.13 ± 0.20^a^	66.14 ± 0.20^a^

^a-e^Means with different superscripts within an extract category differ significantly (*p* ≤ 0.05)

### Sensoric acceptability of functional fish meat product

**Fig. 3:**
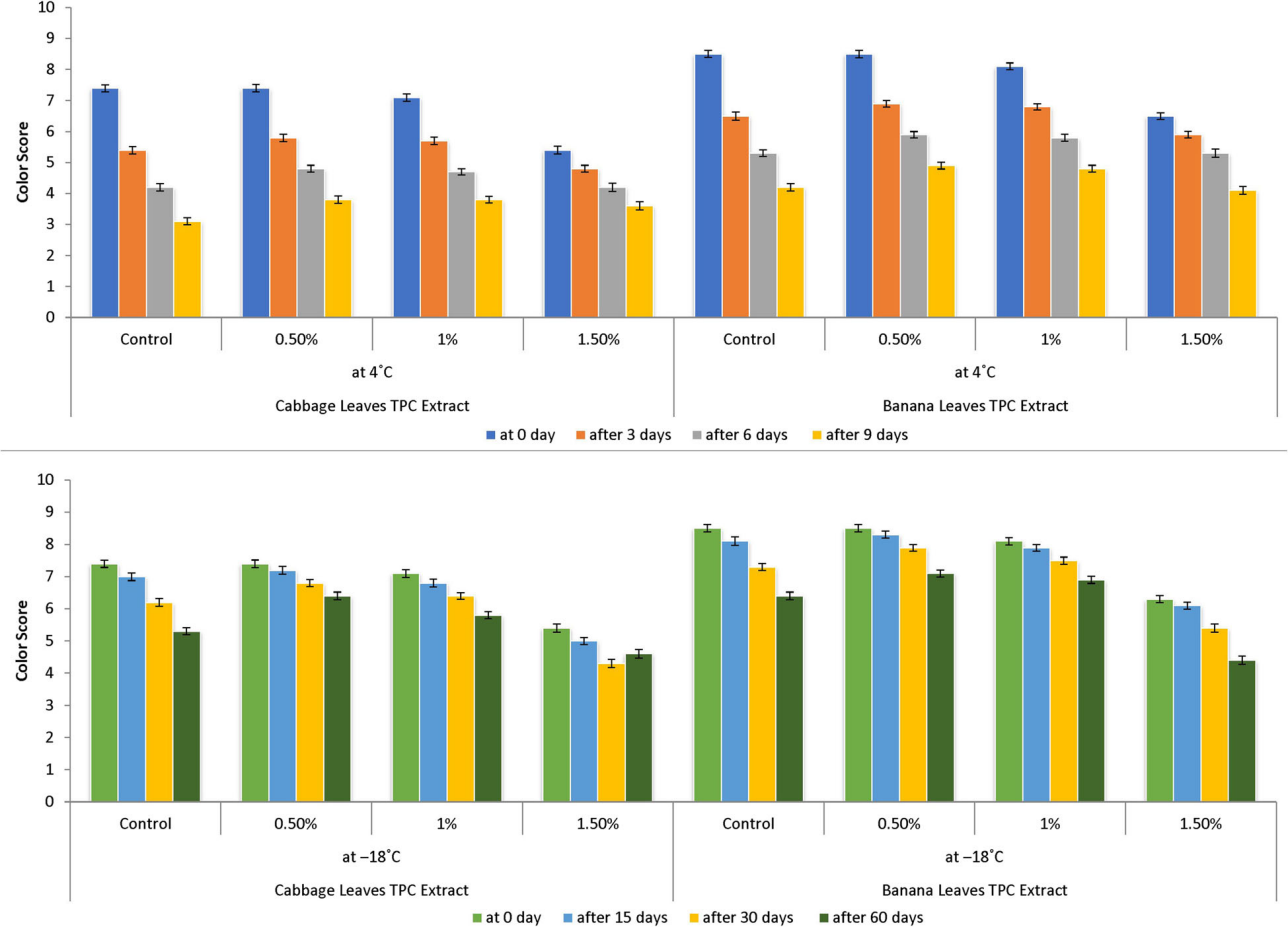
Color evaluation of fish meat balls treated with polyphenol extracts at 4 °C and − 18 °C

**Fig. 4:**
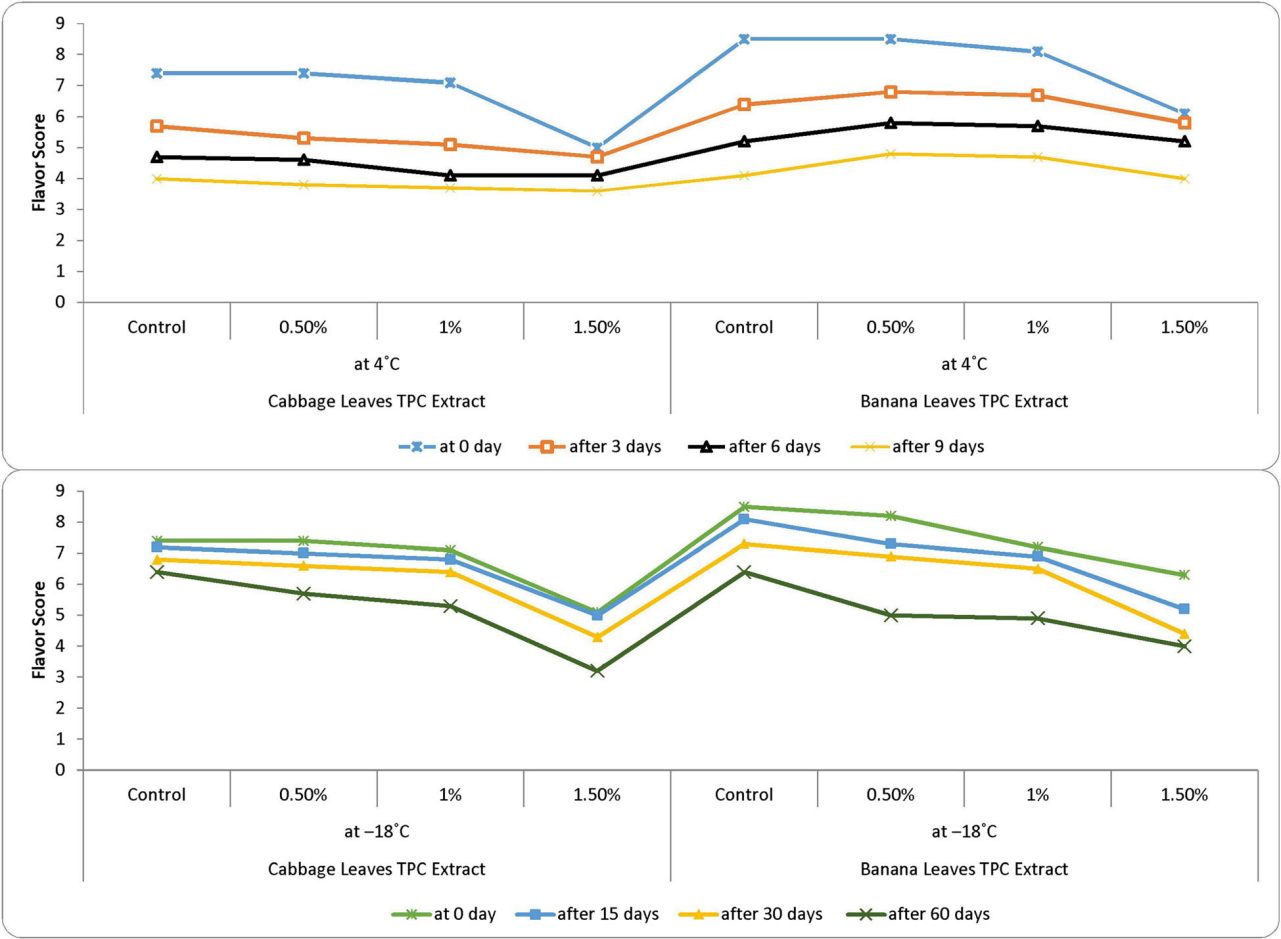
Flavor scores of fish meat balls treated with polyphenol extracts at different storage temperatures and intervals

**Fig. 5:**
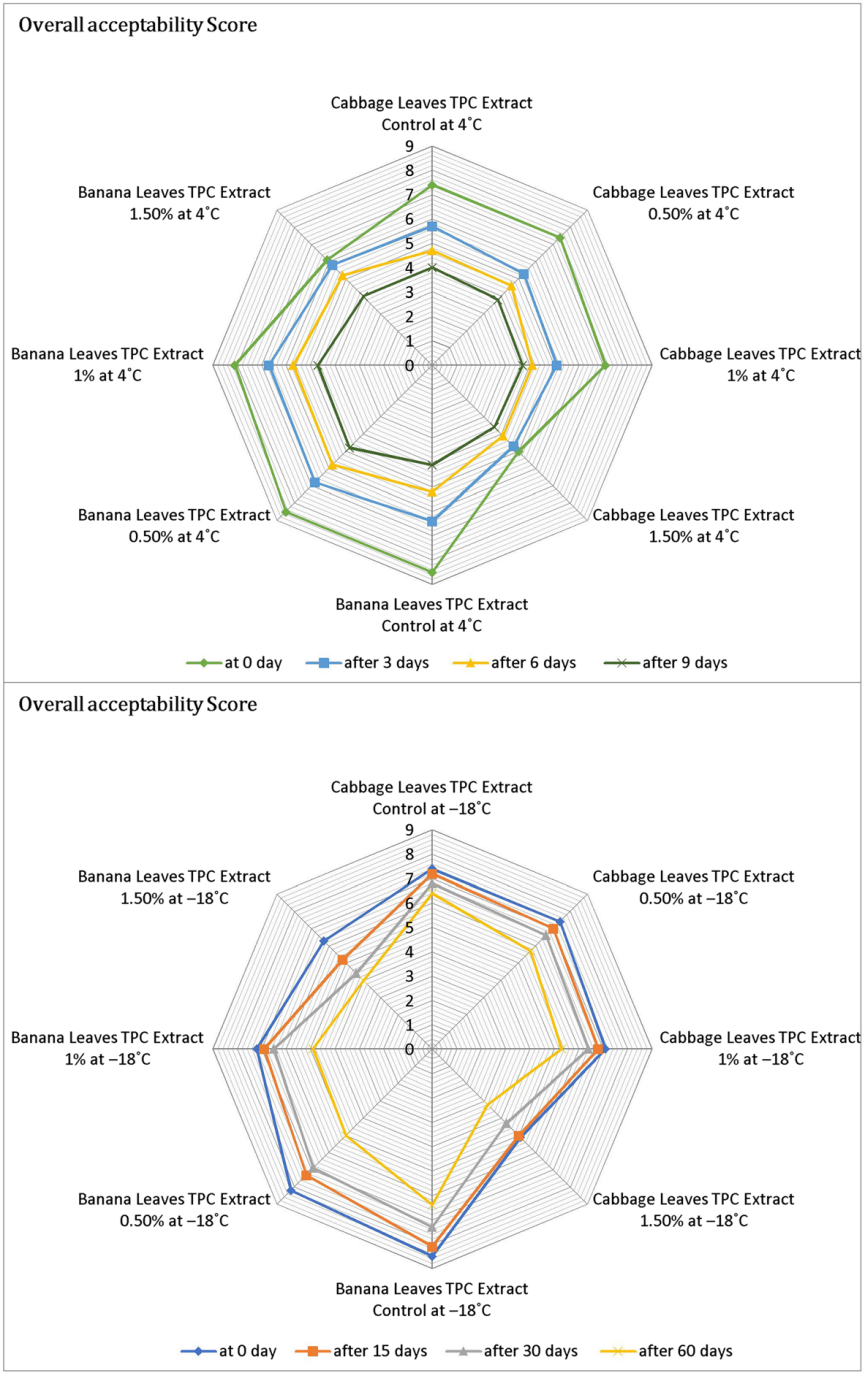
Overall acceptability of fish meat balls treated with polyphenol extracts at 4 °C and − 18 °C during different storage intervals

Sensory evaluation is an important tool in a product development. Acceptance of a food product depends upon the consumer’s perception of the color, taste, texture, flavor and overall acceptability into overall impression of quality. Although, chemical, physical and microbiological tests are employed to check the quality of a food product, but these tests cannot provide such kind of information whether consumer will accept it or not. The meatballs prepared, deep fried and then subjected to sensory evaluation by panelist from parent department for different attributes viz., color, flavor and overall acceptability and scores were recorded using a nine_−_point hedonic scale to assess the liking and disliking of the panelists. Sensory scores of fish product samples for color (Fig. 3), flavor (Fig. 4) and overall acceptability (Fig. 5) show a significant difference in sensory scores at refrigeration temperatures where sensory scores of fish product samples decreased significantly (*p* <  0.05) throughout refrigeration storage. Whereas the sensory scored at the − 18 °C shows the good sensory characteristics, relatively. Acceptable at end of 2nd month with treatment 0.5% results were in parallel to findings of Mahmoudzadeh et al. [37]. When fish-based products are frozen and cold stored, unfavorable changes take place in the texture and appearance. Both FFA and peroxides have been implicated as the cause of decrease in sensory scores [35]. The appearance, texture, odor and flavor of the fish burger started decreasing at a significant level of 1% level [23]. Turhan et al. [38] determined the shelf life of refrigerated raw anchovy (E. encrasicholus) patties for 6 days at 4 °C. The above_−_mentioned studies showed the very similar results with the present study. According to Orak and Kayisoglu [29], the decrease in the values of sensory analyses was faster than chemical changes during frozen storage. Appearance of off_−_flavor may be attributed to WOF (Warm Over Flavor). WOF is usually associated with reheated meats and includes odors and flavors commonly described as stale or rancid also interaction of ketones, aldehydes, alcohols, hydrocarbons, acids, and epoxides with proteins may be produced off_−_colors [39]. Sensory scores decreased during the storage of anchovy patties at the storage temperature of 4 °C and Anchovy patties were consumable up to 6 days [24]. Fish and meat products undergo rapid post_−_mortem changes, mostly proteolysis and lipolysis, which takes product towards sensory deterioration and the risks of food_−_borne illnesses becomes high [40]. Overall, protein and lipid oxidation can account for the toughened texture, off-flavor and unappealing odor of frozen stored sea foods [39].

## Conclusions

Various techniques of food preservation are available either for conventional or modern technology preservation methods. In this regard, the preservation methods used to preserve food should be suitable for the food conditions because not all of these methods are able to maintain the freshness and organoleptic properties of the food products. Natural preservatives have gained interest due to the awareness on the harmful effect of consuming artificial preservatives. It can be concluded that the several fruit and vegetable byproducts from food processing industries meet the criteria of antioxidant definition. They are certain to be an excellent source of natural antioxidants if used as high_−_quality ingredients in functional foods or dietary supplements. The applications of plant food byproducts will definitely bring about added value to both, the industry and the consumer. The industry can benefit from economic incomes and the consumer from the excellent nutritional value of these materials with potential health claims.
